# Evolving synergetic interactions

**DOI:** 10.1098/rsif.2016.0282

**Published:** 2016-07

**Authors:** Bin Wu, Jordi Arranz, Jinming Du, Da Zhou, Arne Traulsen

**Affiliations:** 1 Department of Evolutionary Theory, Max Planck Institute for Evolutionary Biology, August-Thienemann-Straße 2, 24306 Plön, Germany; 2 School of Sciences, Beijing University of Posts and Telecommunications, Beijing 100876, People's Republic of China; 3 Liaoning Key Laboratory of Manufacturing Systems and Logistics, Institute of Industrial Engineering and Logistics Optimization, Northeastern University, Shenyang 110819, People's Republic of China; 4 Center for Systems and Control, College of Engineering, Peking University, Beijing 100871, People's Republic of China; 5 School of Mathematical Sciences, Xiamen University, Xiamen 361005, People's Republic of China

**Keywords:** synergetic interactions, multiplayer games, cooperation

## Abstract

Cooperators forgo their own interests to benefit others. This reduces their fitness and thus cooperators are not likely to spread based on natural selection. Nonetheless, cooperation is widespread on every level of biological organization ranging from bacterial communities to human society. Mathematical models can help to explain under which circumstances cooperation evolves. Evolutionary game theory is a powerful mathematical tool to depict the interactions between cooperators and defectors. Classical models typically involve either pairwise interactions between individuals or a linear superposition of these interactions. For interactions within groups, however, synergetic effects may arise: their outcome is not just the sum of its parts. This is because the payoffs via a single group interaction can be different from the sum of any collection of two-player interactions. Assuming that all interactions start from pairs, how can such synergetic multiplayer games emerge from simpler pairwise interactions? Here, we present a mathematical model that captures the transition from pairwise interactions to synergetic multiplayer ones. We assume that different social groups have different breaking rates. We show that non-uniform breaking rates do foster the emergence of synergy, even though individuals always interact in pairs. Our work sheds new light on the mechanisms underlying such synergetic interactions.

## 1. Introduction

Cooperators benefit others bearing a cost to themselves [1,2]. However, from a Darwinian perspective, cooperation is difficult to explain, as natural selection promotes selfishness rather than cooperation [3–7]. The evolution of cooperation has often been approached through the lens of two-player games that depict the simplest form of social dilemmas [8–10]. The study of games such as the Prisoner's Dilemma, or alternatives such as the Stag-Hunt game [11], has provided insightful views on which mechanisms are likely to promote cooperation—e.g. spatial reciprocity, direct reciprocity, indirect reciprocity, kin selection and group selection [12–14]. However, the simplicity of two-player games is a double-edged sword, as these pairwise games may fail to capture the intricacies of complex interactions in real social and biological systems. Cooperation typically involves multiple interaction partners at the same time rather than a collection of pairwise interactions. For instance, when interacting cells form a multicellular organism, a superposition of pairwise interactions is insufficient to capture the intricacies of the complex organism. This is because an interaction among all the cells is not just a sum of pairwise interactions. Synergetic interactions—the whole being more than the sum of its parts—may be essential to explain cooperation in such biological systems. Therefore, understanding how synergetic interactions emerge is an important part of our understanding cooperation in more realistic interaction scenarios between multiple actors.

**Figure 1.**
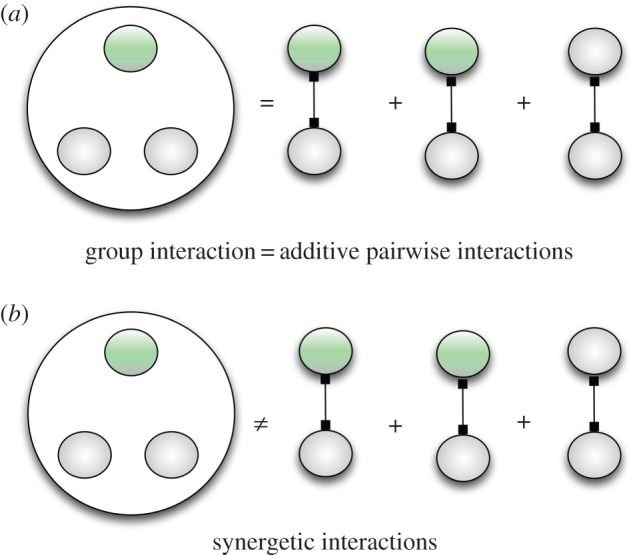
Additive pairwise interactions and synergetic interactions. Additive pairwise interactions mean that a group interaction is equal to the sum of pairwise interactions in payoff. Synergetic interactions refer to the non-equivalence in payoff between the above two ways. For instance, given three individuals in a set, their payoff can be calculated either by three pairwise interactions (right-hand side) or by one group-level interaction (left-hand side). If both methods lead to the same payoff, then the multiplayer game can be expressed as three additive pairwise interactions. As a consequence there is no synergy (*a*). On the other hand, if the payoffs are different, the group-level interaction is not anymore a combination of pairwise games. In this case, a synergetic interaction emerges (*b*).

General multiplayer games, which cannot be decomposed into pairwise interactions, can represent such synergy effects. In this paper, synergetic interactions refer to the non-equivalence between a single interaction as a group (one multiplayer game) and the sum of a number of pairwise interactions (several two-player games; figure 1). A multiplayer game can display broader and richer dynamics than the traditional two-player counterparts [15–22]. In particular, it can exhibit payoff nonlinearities and can thus account for the synergetic effects that are intrinsic to group interactions. Although the emergence of synergetic interactions among multiple players is key to cooperation [16,23], we lack fundamental understanding on how such complex synergetic interactions occur in the first place [24]. The emergence of a matrix multiplayer game, which is directly connected to social dilemmas, is seldomly addressed, but similar questions have been discussed in the context of knockout contests [25,26]. Here, we present a mathematical model that captures the emergence of such synergetic multiplayer games from simple pairwise interactions.

## 2. Results

### 2.1. Model description

**Figure 2.**
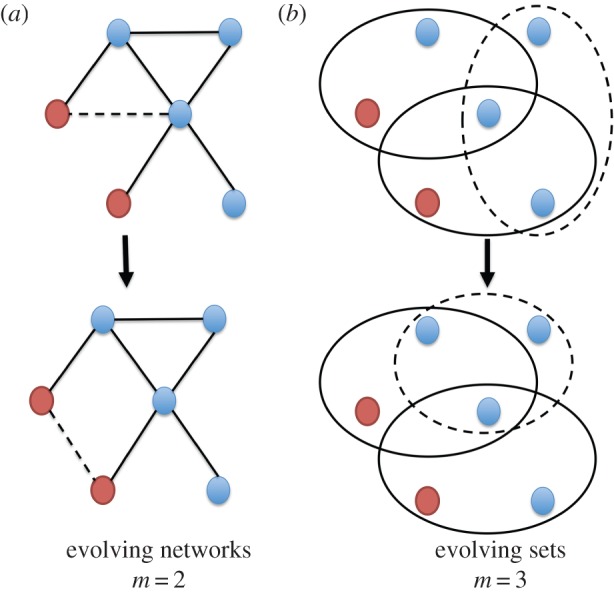
Set dynamics for sets of size *m* = 2 (*a*) and *m* = 3 (*b*). Note that two individuals can interact more than once for sets, which is usually not assumed for networks. Blue and red dots represent strategies *A* and *B* respectively. When *m* = 2 (*a*) ‘sets’ are actually ‘links’ and the overall structure is a network [32]. In this case, interactions are strictly pairwise, hence there is no synergetic effect in the payoffs. (*b*) The case with *m* = 3, which is the minimum set size that illustrates the emergence of synergetic interactions. With probability 1 − *w* a set is selected at random (dashed lines). This set breaks up with probability *k_i_*, where *i* is the number of strategy *A* individuals in the set. If the set breaks, a randomly chosen individual is expelled. In order to keep the size of the set constant, another random individual is incorporated into the updated set (dashed lines).

We consider a structured population of *N* individuals, assorted into *l* sets [27], each consisting of *m* individuals. Individuals can have a different number of set memberships and play one of two strategies, *A* or *B*. An individual accumulates the payoff through interactions within all the sets it belongs to. These interactions are always pairwise and the payoff depends on the set configuration, i.e. the numbers of individuals playing *A* and *B* in the set. At every time-step, either the strategy of an individual or the set structure is updated. With probability *w*, the strategy of an individual is updated. In that case, two individuals are randomly chosen and one imitates the other's strategy with a probability that increases with the payoff difference. Individuals with higher payoffs are more likely to be imitated [28–30]. With probability 1 – *w*, a set is randomly chosen. In that case, the set may break up with probability *k_i_*—where *i* is the number of strategy *A* individuals in the set, ranging from 0 to *m*. As a consequence, *k_i_* determines the fragility of a set, which in turn depends on the set composition [31]. If the set breaks, a randomly chosen individual—which belongs to at least one other set—is expelled. In order to keep the size of the set constant, another random individual is incorporated into the set (figure 2).

Although our model is simple, it captures two fundamental aspects. Firstly, the set structure mimics the social interactions which is an intrinsic characteristic of biological systems and human societies. For instance, the set size could be based on the diffusion rate of the public goods secreted by cooperative cells [33,34]. The overall structure also allows us to consider an organization of arbitrary size, from a small patch to a large assembly. Secondly, individuals only interact in pairs and payoffs are additive. Therefore, the payoff of an individual is nothing but the sum of the corresponding pairwise interactions. Thus, there are no imposed synergetic effects via the payoff accumulation process. Instead, they can only emerge from the dynamics of the population structure.

#### 2.1.1. Pairwise games between two strategies

If the size of the sets is two, *m* = 2, the population structure is equivalent to a network, where a ‘set’ becomes a ‘link’ (figure 2). In this case, the accumulated payoffs can still be captured by a pairwise interaction, hence there are no synergetic multiplayer interactions [32,35]. Although our analytical framework is general enough to allow the study of any set size, we focus for simplicity on the case where *m* = 3, that is, when the sets contain three individuals. Let us assume that for two individuals playing strategy *A*, each one obtains a payoff of *a*. Similarly, for two individuals playing strategy *B*, each one obtains a payoff of *d*. For two individuals playing different strategies, the individual using strategy *A* obtains the payoff *b* and the individual using strategy *B* obtains the payoff *c*. Given that there are three individuals in every set, the payoff of an individual within a set is determined by two pairwise interactions. Therefore, the payoff of an individual playing strategy *A* in a set with *i* other individuals playing *A* is given by 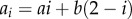. The payoff of an individual playing strategy *B* in a set with *i* other individuals playing *A* is given by 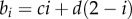. Note that for *m* = 3, *i* = 0, 1, 2. Given that the breaking probability of a specific set may depend on the number of *A* individuals within the set, the set dynamics allows for non-uniform breaking probabilities across the sets.

To demonstrate that our simple model can indeed capture the emergence of synergy, we consider two aspects: the accumulated payoff of both types and the evolutionary dynamics between the two strategies. We find that non-uniform breaking probabilities across the sets foster the emergence of synergetic multiplayer interactions. In other words, when the fragilities of the sets are non-uniform we find that (i) the expected accumulated payoff of both strategies is consistent with the one of a typical multiplayer game that cannot be decomposed into a pairwise game and (ii) the evolutionary dynamics of the strategies exhibit two internal equilibria of selection, which is impossible in a two-player game.

The calculation of the average accumulated payoff in the general case is challenging, even though the model is simple. We overcome this problem by assuming that the probability with which the strategy is updated is small, 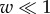. This could for example be the case in microbial communities with scarce resources. The mobility rate is high in this case, resulting in a large number of structural changes for each reproduction event. As a consequence, the set structure can reach its stationary state—which determines the accumulated payoffs—before a strategy update occurs. Importantly, the average accumulated payoffs for both strategies are consistent with the payoffs of the following 3-player game in a well-mixed population, up to a positive rescaling factor (see the electronic supplementary material, appendix, §2.1):2.1
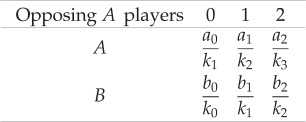
Here 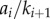 is the payoff for an individual using strategy *A* when it meets *i* opponents using strategy *A*. Equivalently, *b_i_/k_i_* is the payoff for an individual using strategy *B* when it meets *i* opponents using strategy *A*. The payoff table in equation (2.1) has two important features. First, the derived multiplayer game is of the same size as that of the set, which implies that the complexity of the game is naturally bounded by the set size. Second, the payoff entries are proportional to the product of the accumulated payoff in a set and its lifetime.

The evolutionary outcome of both strategies can be calculated by the replicator equation if the population size is sufficiently large (see the electronic supplementary material, appendix, §2.2) [36]. Qualitatively, this outcome would also arise for a large number of microscopic update mechanisms [30,37]. The replicator equation is given by2.2
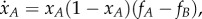
where2.3
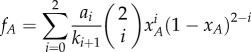
2.4
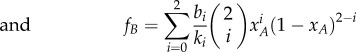
are the average accumulated payoffs for strategy *A* and *B*. Here, *x_A_* is the fraction of individuals using strategy *A*. On the one hand, intuitively, an individual playing strategy *A* obtains 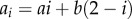 via two pairwise interactions in a set consisting of *i* other strategy *A* players (*i* = 0, 1, 2). The probability of finding the set consisting of *i* other strategy *A* player is proportional to 
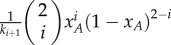
. Here, 1/*k_i_*_+1_ is the average duration time of the sets consisting of *i* + 1 strategy *A* individuals. Thus, the average accumulated payoff of strategy *A* individual is given by the weighted sum of *a_s_*, which leads to *f_A_*, i.e. equation (2.3). On the other hand, equation (2.3) can be interpreted in another way: it represents the payoff of the strategy *A* individuals of the 3-player game based on equation (2.1) in a well-mixed population. An equivalent argument applies for the average accumulated payoff of strategy *B*, i.e. equation (2.4). Therefore, the dynamics of the pairwise game under active set dynamics can be captured by a multiplayer game in a well-mixed population. The internal equilibria of this equation are the roots of the equation *f_A_* − *f_B_* = 0. Based on the initial fraction of individuals using strategy *A*, these equilibria determine where an infinite population would end up, depending on the initial fractions of each strategy.

The above results on the accumulated payoffs and the evolutionary dynamics of strategies hold for any set fragilities 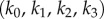. In the following, we apply these results to homogeneous and heterogeneous set fragilities to address when and how synergetic interactions emerge.

**Figure 3.**
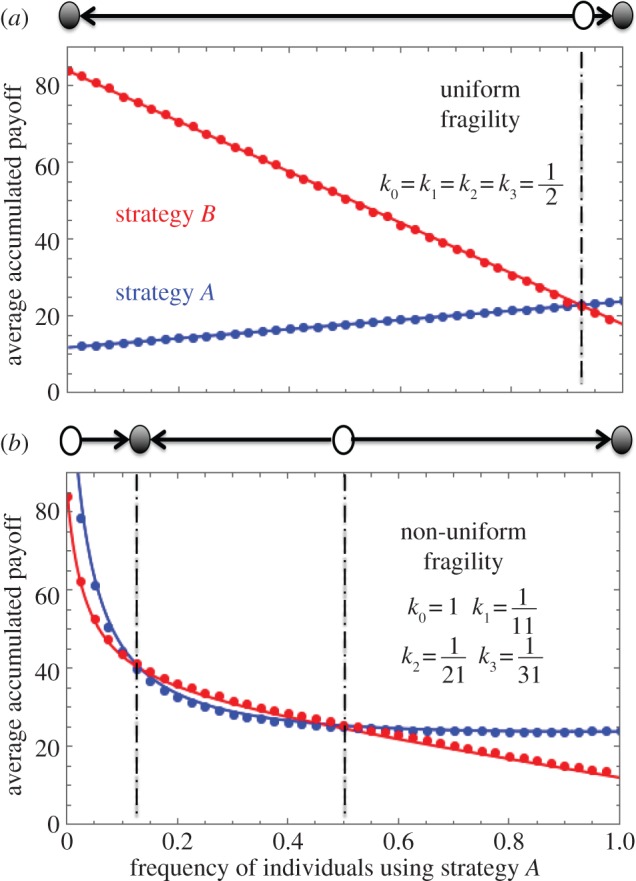
Accumulated payoffs for the Stag-Hunt game and the equilibria of the selection gradient for two fragility scenarios. The full/empty circles on top of each panel show the analytical approximation for the stable/unstable equilibria of the replicator dynamics. (*a*) For uniform breaking rates, the accumulated payoffs for both strategies match the ones of a pairwise game. Thus, the payoffs change linearly with the fraction of *A* individuals and the replicator equation predicts just the single internal equilibrium of the pairwise game. (*b*) When the breaking rates depend on the set configuration, the payoffs for both strategies become nonlinear. The more *A* individuals a set has, the less likely it breaks up. The payoffs for both strategies have two intersections, which lead to two internal equilibria. This illustrates that non-uniform interactions can lead to the emergence of synergetic interactions. There is a perfect agreement between simulations (dots) and analytical approximation (lines). (Parameters: Stag-Hunt game with *a* = 2, *b* = 1, *c* = 1.5 and *d* = 7. Population size, *N* = 500, number of sets, *l* = 1000, probability of a strategy update, *w* = 10^−3^. Selection intensity, *β* = 0.1. See Methods for the simulation details.)

Whenever the fragility of the sets is constant, 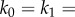 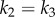, *f_A_* is proportional to 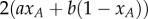 based on equation (2.3). This is nothing but the sum of two pairwise interactions. In this case, all the sets have the same duration. This suggests that a random sampling of sets leads to a well-mixed like population structure. Therefore, the average accumulated payoff of strategy *A* is the sum of payoffs arising from the two pairwise interactions. In other words, equation (2.1) is identical to the original pairwise game, even though it is formally a 3-player game (see the electronic supplementary material, appendix, §2.2). Therefore, there is no synergy effect in payoff. Given that the replicator equation is equivalent to the one of the pairwise game, there is at most one internal equilibrium with the same position and stability. Figure 3*a* shows the agreement between the analytical approximation and a simulation of the full model.

**Figure 4.**
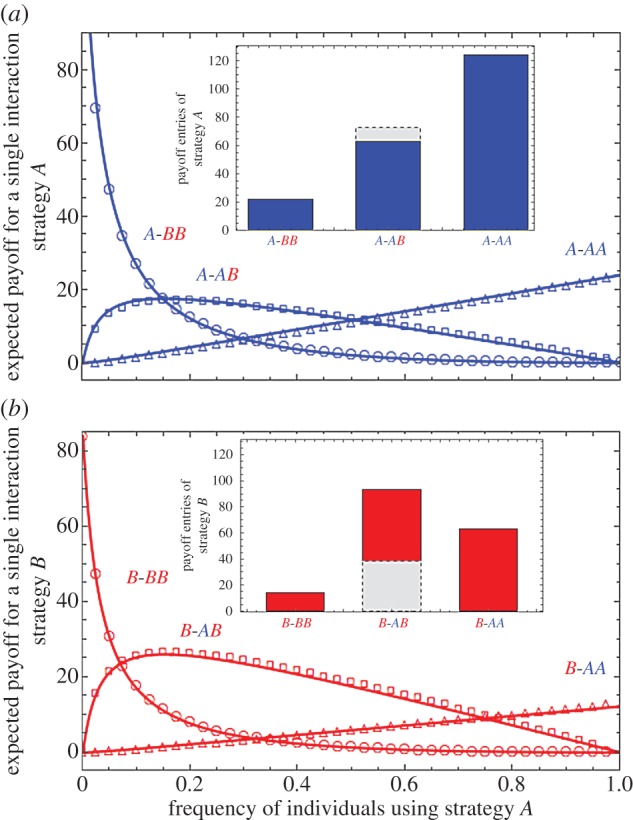
Expected payoffs for individuals using strategies *A* (*a*) and *B* (*b*) in groups of different compositions. The two insets within each plot show the effective payoff entries of the emergent 3-player game equation (2.1) for a frequency of 0.5. Main panels: theoretical predictions for the payoff within the three set configurations of each individual, equations (2.3) and (2.4) (lines), agree well with the accumulated payoff obtained by simulation (symbols). This in turn proves that the synergetic three-player game is intrinsically captured by equations (2.3) and (2.4) even term by term. (*a*) Inset: payoffs for equal abundance of both strategies. An individual with strategy *A* gains less if it interacts in a set which has one individual with strategy *A* than if it were in the synergy free case (grey, dashed). (*b*) Inset: an individual with strategy *B* gains much more if it interacts in a set which has one individual with strategy *A* than if it were in the synergy free case (grey, dashed). Here, the payoffs in the synergy free cases are the average values of the two payoff entries for the focal individual interacting with zero and two *A* individuals. Thus, the interaction can no longer be decomposed into multiple pairwise interactions, which is how every individual obtains its payoff microscopically. (Parameters are the same as that in figure 3*b*. The inner panels are obtained by simulation via setting the frequency of individuals using strategy *A* to be one half. See Methods for simulation details.)

However, when fragilities are not homogeneous across the sets, equation (2.1) becomes a 3-player game, which cannot be decomposed into additive pairwise interactions (figure 3*b*). In this case, the payoff of an individual interacting with two opponents is not equal to the sum of the two pairwise interactions (figure 4). The intuition is given as follows: let us assume that the strategy *B* individuals are very likely to leave the sets when the other co-players in the set are all of strategy *B* (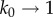). In this case, almost all the payoffs strategy *B* individuals obtain are via the set configurations where there is at least one strategy *A* individual. The short duration of pure *B* sets leads to the effective payoff of strategy *B* individuals in a set consisting of two-strategy *B* co-players to be approximately zero. On the other hand, once there is such a set, a strategy *B* individual gets 2*d* via two pairwise interactions. The inconsistency between zero and 2*b* emerges from non-uniform interactions. Consequently, the presence of non-uniform set breaking probabilities generates synergetic payoffs. Synergy emerges exclusively as a result of the evolutionary dynamics of the set structured population. In addition to this, the replicator equation has two internal equilibria, which is not possible in pairwise interactions (figure 3*b*). A necessary condition for the emergence of two equilibria is that the sign of the effective payoff difference 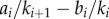 changes twice with the increase in the number of opponents using strategy *A*, *i* [38,39]. A more detailed analysis of the conditions that lead to two internal equilibria can be found in the electronic supplementary material, appendix, §2.2. If one of the two equilibria is stable, the other has to be unstable. Given this, our model can explain both the maintenance of biodiversity and phenotypic dominance within the same framework, despite being composed of pairwise interactions which do not lead to these phenomena *per se*.

#### 2.1.2. Pairwise games between *n* strategies

The model can be extended to account for an arbitrary number of strategies, *n*, instead of only two. In the pairwise interactions with *n* strategies or an *n* × *n* game, the non-uniform breaking probabilities also generate synergetic multiplayer interactions. Although the analytical calculations are more intricate due to the increased number of set configurations, we find that the payoff matrix of the emergent multiplayer game is consistent with the one of an *n*-strategy *m*-player game (electronic supplementary material, appendix, §3.1). Interestingly, the intuition behind these payoff entries is the same as the ones of the two-strategy case, as they still represent the product of the additive payoffs via pairwise interactions and the duration of that set. In addition to this, the *n*-strategy *m*-player game has, at most, (*m* − 1)*^n^*^−1^ isolated internal equilibria, whereas the original *n* × *n* pairwise game has at most one isolated internal equilibrium (electronic supplementary material, appendix, §3.2). The dynamics in our model are thus rich enough to capture complex phenomena exhibited by social and biological systems.

## 3. Discussion

Synergy refers to the idea that the whole is greater than the sum of its parts. Interestingly, the word *synergy* means ‘cooperation’ in ancient Greek. In the simplest case of constant selection, payoffs are fixed and not dependent on any interactions. Departing from this, one usually considers pairwise games, which lead to a linear dependence of payoffs on the relative abundance of strategies. This linear dependence is also reflected in linear multiplayer games, which can thus be decomposed into a collection of pairwise games. Here, we explicitly consider multiplayer games, where generically payoff becomes a polynomial function of the relative abundance of strategies. In particular, we are interested in whether the payoff of a multiplayer game is different from the outcome of the sum of the underlying pairwise interactions (figure 1). If this is the case, we speak of the emergence of synergetic interactions among multiple players.

Synergetic interactions are found in a plethora of different contexts such as in genes [40], microbial populations [41], and even social and economic systems. With this in mind, we present a minimal model that shows how synergetic interactions can emerge in a system based on pairwise interactions. An example for such a situation is the production of extracellular products by cooperating cell types, which glue individuals together; see [42] for a related theoretical analysis. This glue production would increase the lifetime of cooperator groups compared to groups with few cooperators. However, our model is intended as a minimal way to showcase the emergence of multiplayer interactions and not as a model of a particular biological instance.

From an evolutionary perspective, synergy is a cornerstone of fraternal evolutionary transitions [43]—e.g. the emergence of multicellularity [1]. The non-uniform breaking rates which are a feature of our model can mimic the migration rates of cells in different environments. This leads to a synergetic interaction where a group of cells perform differently from the sum of cell–cell interactions. Given this, it appears that fitness differences—not driven by group size, but by group composition—could be a key to understand how multicellular organsims perform as a whole.

It is worth highlighting that the sets in the model are not proxies for a new level of selection, but represent the interaction range of individuals. Owing to these restrictions, our approach is not a suitable model for egalitarian and filial transitions [44]. Those transitions involve individuals of different species and therefore require asymmetric games [36].

On the other hand, our model does allow one to explore the emergence of synergetic interactions from pairwise interactions analytically. We assume that the strategy of the individuals and the interaction structure change over in time. The results show that non-uniform set breaking rates, which depend exclusively on the configuration of these sets, lead to payoff nonlinearities. These are consistent with the dynamics of a multiplayer game, even though individuals always play a pairwise game and no group selection effects are present. These findings rely on two conditions: (i) sets must contain more than two individuals and (ii) the breaking rates must depend on the configuration of the sets, and hence be non-uniform.

In our model, individuals interact in sets. The set structure is a generalization of network structure. In that case, individuals interact in groups with more than two members. This resembles microbial communities in biology or clubs and families in human societies. Thus, a set is a more general assumption than a network (figure 2). Furthermore, individuals in our model are allowed to adjust both their strategies and their partners. This mechanism proves to be an efficient way to promote and stablize cooperation in networks with pairwise interactions [24,35,45–51]. Consistent with those studies, our dynamical set model can yield an assortment of cooperators. This paves the way not only to resist the invasion of defectors, but also to invade defectors. A mathematical limitation of our work is the assumption of the fast set dynamics. With this approximation, our model cannot be applied to settings such as microbial communities with abundant resources. In that case, the mobility rate is typically low, resulting in a very small number of structural changes for each reproduction event.

Our model shows how the aggregation of individuals can lead to complex interactions that cannot be disentangled into simpler interactions anymore. The natural world has many examples for such situations, where a complexity can no longer be decomposed.

## 4. Methods

### 4.1. The Fermi updating rule

We use the Fermi update rule, given by the following algorithm [28,29,52]:

(i) randomly select a focal individual, f*, and denote its payoff as 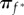;
(ii) randomly select another individual as a role model, r*, among all the individuals in the sets individual f* is in and denote the payoff of r* as 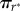; and
(iii) f* switches to the strategy of r* with probability 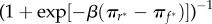.

### 4.2. Accumulated individual payoffs

Each data point is the average of 100 independent realizations. Every realization takes 10^6^ generations. In each realization, for the first 10^4^ generations, only set dynamics occurs to start from a natural set configuration. For the remaining generations, at every step, with probability *w* = 10^−3^ we compute the average accumulated payoff of each strategy. Otherwise, with probability 1 – *w*, set dynamics happens. At the end of each realization, we compute the mean value of all the average accumulated payoff. This allows us to calculate average payoffs in an equilibrium of the set structure for a given abundance of each strategy.

### 4.3. Accumulated group payoffs

Each data point is the average of 100 independent realizations. Every realization takes 10^6^ generations. In each realization, for the first 10^4^ generations, only set dynamics occurs. For the remaining generations, at every step, with probability *w* = 10^−3^ we compute the average accumulated payoff of each strategy within a set with 0, 1 and 2 strategy *A* opponents. Otherwise, with probability 1 − *w*, set dynamics happens. At the end of each realization, we compute the mean value of all the average accumulated payoff.

## Supplementary Material

Supplementary Information of “Evolving synergetic interactions”
