# Supplementary material for: Evolving synergetic interactions
Source: J R Soc Interface. 2016 Jul;13(120):20160282. doi: 10.1098/rsif.2016.0282 (PMC4971219; doi:10.1098/rsif.2016.0282)
Supplement: Supplementary Information of “Evolving synergetic interactions” [file rsif20160282supp1.pdf]

Supplementary Information of "Evolving synergetic interactions"

Bin Wu[1,2], Jordi Arranz[1], Jinming Du[3], Da Zhou[4] and Arne Traulsen[1]
[1] Department of Evolutionary Theory, Max Planck Institute for Evolutionary Biology, August-Thienemann-Straße 2, 24306 Plön, Germany,
[2]School of Sciences, Beijing University of Posts and Telecommunications, Beijing 100876, China
[3] Center for Systems and Control, College of Engineering, Peking University, Beijing 100871, China
[4] School of Mathematical Sciences, Xiamen University, Xiamen 361005, China

June 30, 2016

Contents

1 Evolutionary dynamics in a set structured population **2**

2 Games with two strategies **3**

2.1 Accumulated payoffs 3

2.2 Evolutionary dynamics of strategies 6

3 Games with n strategies **8**

3.1 Accumulated payoffs 8

3.2 Evolutionary dynamics of strategies 12

1 Evolutionary dynamics in a set structured population

Consider a set structured population of fixed size N where individuals engage in a pairwise game. The population is divided into a constant number of sets, l, each of them of constant size m. Individuals can belong to different sets.

At each time step, we either update the strategy of an individual — with probability w — or the structure of the population, with probability $1 - w$.

A strategy update involves two randomly selected individuals, say Alice and Bob. Alice imitates Bob's strategy with a probability depending on the difference of their payoffs, i.e., the imitation rule (1). The payoff of an individual is calculated as the sum of all the pairwise interactions through all the sets it belongs to. For instance, if Alice belongs to two sets, her payoff is the sum of the payoff in the first set, plus the payoff in the second one. Given that every set consists of m individuals, the payoff of an individual in a set is the sum of $m - 1$ pairwise interactions. On the other hand, when set dynamics occur, a set is randomly selected. This set may break with a probability which depends on the set composition. In particular, if there are only two strategies, the set composition is the number of individuals within the set playing a specific strategy. If the set is broken, a random individual within the set is expelled, provided it is in at least one other set. In order to keep the size of the set constant, another random individual is added to the focal set.

We start with the simplest pairwise games with two strategies, A and B. The 2×2 payoff matrix is given by $(a_{ij})_{2\times 2}$, where a_{ij} is the payoff of an individual playing strategy i with an opponent playing strategy j, where $i, j \in \{A, B\}$. We find that the average accumulated payoff for each strategy is consistent with the one of a 2-strategy, m-player game up to a positive rescaling factor. When the breaking probability of a set is uniform across all kinds of sets, the payoff of the m-player game is equivalent to that of a sum of $m - 1$ pairwise games. However, we notice that whenever the sets have different breaking probabilities that depend on the set composition, intrinsic multiplayer interactions emerge. In this case, the accumulated payoff of both strategies cannot be decomposed into collections of pairwise games anymore. In other words, synergetic effects in payoff can emerge from simple pairwise interactions. Based on accumulated payoffs, we further obtain the replicator equation of the m-player game to determine the evolutionary fate of each strategy. In addition to this, the replicator equation shows up to $m - 1$ internal equilibria. In contrast, for pairwise interaction there is at most one such equilibrium. These results are obtained under the assumption of fast set dynamics — very few strategy updates occurs before

the population structure has reached the stationary state — and a large population size.

We generalise the above results for cases where the number of strategies, n, is greater than two. In this case the payoff matrix is given by $(a_{ij})_{n\times n}$, where $i, j \in \{1, 2, \dots, n\}$. In this technically somewhat more challenging case, we find similar results:

(i) The average accumulated payoff of each strategy is of the form of an n-strategy, m-player game up to a positive rescaling factor.

(ii) When the set breaking probabilities are uniform, the payoff of the n-strategy m-player game is still consistent with the sum of the $m-1$ pairwise games.

(iii) Non-uniform set breaking probabilities foster the emergence of multiplayer interactions, which cannot be decomposed into a collection of pairwise games.

(iv) The replicator equation of the n-strategy m-player captures evolutionary dynamics of the strategies and displays, at most, $(n-1)^{m-1}$ internal equilibria, whereas pairwise $n \times n$ games display, at most, a single equilibrium (2).

2 Games with two strategies

2.1 Accumulated payoffs

Initially, i.e. at time $t = 0$, we call the l sets T_i^0, $1 \le i \le l$. For the first time step in the set evolution, $t = 1$, we denote the selected set as $T_{i^*}^0$. If this set is broken and transforms to another set, we denote the transformed set as $T_{i^*}^1$, otherwise the set is not broken and we let $T_{i^*}^1 = T_{i^*}^0$. For the other sets which are not selected, we denote $T_i^1 = T_i^0$, $i \neq i^*$. Recursively, we define T_i^t for $t \ge 0$ and $1 \le i \le l$.

Let $\psi(T_i^t)$ be the number of strategy A individuals in set T_i^t, thus $0 \le \psi(T_i^t) \le m$. For each set i, the dynamics of $\psi(T_i^t)$ is a Markov chain in state space $\{0, 1, 2 \cdots m\}$ with the transition matrix

$$Q = \frac{1}{l}V + \frac{l-1}{l}I_{m+1}, \qquad [1]$$

where I_{m+1} is the identity matrix of size $m+1$ and V is the transition matrix when set i is selected.

Hence, $V = (V_{ij})_{(m+1)\times(m+1)}$ $(0 \le i, j \le m)$ is a tridiagonal matrix given by

$$V_{ij} = \begin{cases} k_i \frac{m-i}{m} x_A & \text{if } j = i+1 \\ 1 - k_i \left(\frac{m-i}{m} x_A + \frac{i}{m} x_B\right) & \text{if } j = i \\ k_i \frac{i}{m} x_B & \text{if } j = i-1 \\ 0 & \text{otherwise} \end{cases}. \qquad [2]$$

Here x_A and $x_B = 1 - x_A$ are the fractions of strategy A and B in the population, k_i is the breaking probability of a set consisting of i individuals playing strategy A and $m - i$ individuals playing strategy B.

When $x_A > 0$ and $x_B > 0$, the matrix Q is irreducible and aperiodic and there is a unique stationary distribution $y = (y_0, y_1, \cdots, y_m)$ of Q determined by $yQ = y$. Taking Eq. (1) into $yQ = y$ leads to $yV = y$. This leads to the stationary distribution

$$y_s = \frac{1}{\mathcal{N} k_s} \binom{m}{s} x_A^s x_B^{m-s}, \ \ 0 \le s \le m, \qquad [3]$$

where $\mathcal{N} = \sum_{s=0}^{m} \binom{m}{s} \frac{1}{k_s} x_A^s x_B^{m-s} > 0$ is a normalisation factor. The stationary distribution also represents the proportion of each type of set among all the sets in the stationary regime.

When set dynamics are fast, the imitation event happens rarely enough to allow the population structure to reach the stationary state before a single imitation event occurs. In this case, the stationary regime of the set dynamics determines the average payoff of both strategies. Here the accumulated payoff of strategy A is given by

$$\begin{aligned} f_A &= \frac{\text{Total payoff of all the strategy } A \text{ individuals}}{\text{Number of strategy } A \text{ individuals}} \\ &= \frac{\sum_{j=1}^{m} l y_j j (a_{AA}(j-1) + a_{AB}(m-j))}{N x_A} \\ &= \frac{1}{\mathcal{N}} \frac{l}{N} \sum_{j=1}^{m} \binom{m}{j} j \frac{1}{k_j} x_A^{j-1} x_B^{m-j} \underbrace{(a_{AA}(j-1) + a_{AB}(m-j))}_{a_{j-1}}. \end{aligned} \qquad [4]$$

As $j\binom{m}{j} = m\binom{m-1}{j-1}$, Eq. (4) can be written as

$$\frac{1}{\mathcal{N}} \frac{lm}{N} \sum_{j=1}^{m} \binom{m-1}{j-1} x_A^{j-1} x_B^{m-j} \frac{a_{j-1}}{k_j}. \qquad [5]$$

With $s = j - 1$, Eq (5) becomes

$$f_A = \frac{1}{\mathcal{N}} \frac{lm}{N} \underbrace{\sum_{s=0}^{m-1} \binom{m-1}{s} x_A^s x_B^{m-1-s} \frac{a_s}{k_{s+1}}}_{\tilde{f}_A}. \quad [6]$$

Similarly, we have

$$f_B = \frac{1}{\mathcal{N}} \frac{lm}{N} \underbrace{\sum_{s=0}^{m-1} \binom{m-1}{s} x_A^s x_B^{m-1-s} \frac{b_s}{k_s}}_{\tilde{f}_B}, \quad [7]$$

where $b_s = a_{BA}s + a_{BB}(m-1-s)$ is the accumulated payoff of an individual using strategy B gets in a set consisting of s individuals using strategy A.

Besides the common positive rescaling factor $\frac{1}{\mathcal{N}} \frac{lm}{N}$, the average accumulated payoffs for both strategies are $\tilde{f}_1$ and $\tilde{f}_2$. Interestingly $\tilde{f}_A$ and $\tilde{f}_B$ are exactly the payoff of an m-player game in a well-mixed population,

Opposing strategy A players	0	$\ldots$	s	$\ldots$	$m-1$
Strategy A	a_0/k_1	$\ldots$	a_s/k_{s+1}	$\ldots$	a_{m-1}/k_m
Strategy B	b_0/k_0	$\ldots$	b_s/k_s	$\ldots$	b_{m-1}/k_{m-1}

. [8]

Here a_s/k_{s+1} (b_s/k_s) is the payoff of an individual using strategy A (B) obtains when it meets s strategy A opponents. This payoff table has two remarkable features: First, the number of players in the multiplayer game and the set size are equal. Second, the payoff entries of the multiplayer game are the product of the linear collective payoff via pairwise interactions in a set and the duration of the corresponding set. Taking a_0/k_1 as an example, a_0/k_1 is the payoff of an individual playing strategy A when he interacts with 0 strategy A opponents, or $m-1$ strategy B individuals. However, within a set, the accumulated payoff of an individual playing strategy A is given by $a_0 = (m-1)a_{12}$ and results from the $m-1$ pairwise interactions in the set where $1/k_1$ is the duration of the set. In other words, a_0/k_1 is the accumulated payoff rescaled with the interaction time.

When $m = 2$, the population strcture is equivalent to a network and the sets represent links. In this case, the effective payoff table in Eq. (8) is still a pairwise game. This transformation can alter the effective payoff of both strategies, however it cannot lead to synergetic effect in payoffs as there is only one pairwise interaction in the transformed table. When $m = 3$, the emergent payoff table is consistent with a 3-player game. On one hand, we can take the payoff entries as the synergetic payoff of two individuals. On the other hand, we have the additive payoffs via the two pairwise interactions in that set. A comparison between these two payoffs can facilitate us to study when "the whole is better than the sum of its parts", i.e., the synergetic payoff is better off than that derived by two pairwise interactions.

When the set breaking probabilities are uniform, i.e., k_i is constant, we find — by Eq. (6) and Eq. (7) — that the accumulated payoffs for strategy A and B are $f_A = (m-1)(a_{11}x_A + a_{12}x_B)$ and $f_B = (m-1)(a_{21}x_A + a_{22}x_B)$. That is to say that the emergent payoff is equivalent to the sum of the corresponding $m-1$ pairwise game and therefore there is no synergy. However, when the set breaking probabilities are non-uniform, intrinsic multiple player games emerge. In this case the "whole" is different from the sum of its parts.

2.2 Evolutionary dynamics of strategies

In large well mixed populations, the evolutionary dynamics of strategies based on the imitation rule can be approximated by the Langevin equation (3)

$$\begin{aligned} \dot{x_A} &= x_A(1-x_A)\left(g(\beta(f_A - f_B)) - g(\beta(f_B - f_A))\right) \\ &\quad + \sqrt{\tfrac{x_A(1-x_A)(g(\beta(f_A-f_B))+g(\beta(f_B-f_A)))}{N}}\xi. \end{aligned} \qquad [9]$$

Here ξ is the white Gaussian noise, f_A (Eq. 6) and f_B (Eq. 7) are the average accumulated payoffs for strategy A and B, respectively. In addition, g is the imitation function capturing the likelyhood of the focal individual to adopt the strategy of the opponent's and β is the selection intensity (4). Throughout, g' is positive, implying that individuals are likely to adopt the strategy of individuals with high payoffs. In particular, the Fermi update rule is an imitation update rule with the imitation function $g(x) = [1 + \exp(-x)]^{-1}$.

For large population size N, the stochastic term vanishes and we obtain

$$\dot{x}_A = x_A(1-x_A)\left(g\left[\frac{1}{\mathcal{N}}\frac{lm\beta}{N}(\tilde{f}_A - \tilde{f}_B)\right] - g\left[\frac{1}{\mathcal{N}}\frac{lm\beta}{N}(\tilde{f}_B - \tilde{f}_A)\right]\right). \quad [10]$$

Note that $\frac{1}{\mathcal{N}}\frac{lm\beta}{N}$ is always positive and $g' > 0$, the equilibria of this equation are the same as that of the following replicator equation of the multiplayer game Eq. (8) in position and stability

$$\dot{x}_A = x_A(1-x_A)(\tilde{f}_A - \tilde{f}_B). \quad [11]$$

Therefore, the evolution of a pairwise game on the evolving set structured population is captured by an m-player game in a well mixed population. Under uniform breaking probabilities, the replicator equation Eq. (11) is consistent with the one of the pairwise game in well-mixed population. At most one internal equilibrium can arise in this case. Under non-uniform breaking probabilities, however, Eq. (11) can exhibit up $m-1$ internal equilibria.

For any set size m, the internal roots of the replicator equation are determined by the roots of the following Bernstein polynomial (5)

$$\tilde{f}_A - \tilde{f}_B = \sum_{s=0}^{m-1} \underbrace{\left(\frac{a_s}{k_{s+1}} - \frac{b_s}{k_s}\right)}_{\Delta d_s} \binom{m-1}{s} x^s (1-x)^{m-1-s} = 0, \quad [12]$$

where $x \in (0,1)$. By the variation diminishing property (6) we know that the number of the internal roots is equal to the number of sign changes of $(\Delta d_0, \Delta d_1 \cdots, \Delta d_{m-1})$, or less by an even number. In particular, when there is only one sign change in the sequence $(\Delta d_0, \Delta d_1 \cdots, \Delta d_{m-1})$, there is exactly one internal equilibrium.

When the set size m is 3, there can be, at most, two internal equilibria (Figure. 1). A necessary condition for the existence of two equilibria is either $\Delta d_0 > 0$ $\Delta d_1 < 0$ and $\Delta d_2 > 0$, or $\Delta d_0 < 0$ $\Delta d_1 > 0$ and $\Delta d_2 < 0$. In both cases, the sign of the coefficient changes twice. The variation diminishing property tells us that there can be either two or no internal equilibria. Since $\Delta d_0 > 0$ $\Delta d_1 < 0$ and $\Delta d_2 > 0$ is equivalent to $\Delta d_0 < 0$ $\Delta d_1 > 0$ and $\Delta d_2 < 0$ by exchanging the name of the two strategies. We focus on $\Delta d_0 < 0$ $\Delta d_1 > 0$ and $\Delta d_2 < 0$. In this case, the Bernstein polynomial Eq. (12) is negative at $x = 0$ and 1. Thus, the existence of two internal equilibria is equivalent to that the maximum of the Bernstein polynomial in $(0,1)$ has to be positive. In the present case, the Bernstein polynomial is quadratic with a maximum at $x^* = (\Delta d_1 - \Delta d_2)/((\Delta d_1 -$

$\Delta d_2) + (\Delta d_1 - \Delta d_0)) \in (0, 1)$. Therefore, the Bernstein polynomial is positive at x^* if

$$-2\Delta d_0 \Delta d_1 \Delta d_2 + 2(\Delta d_1)^3 - \Delta d_0(\Delta d_1)^2 - \Delta d_2(\Delta d_1)^2 + \Delta d_0(\Delta d_2)^2 + \Delta d_2(\Delta d_0)^2 > 0 \qquad [13]$$

To sum up, there are two internal equilibria if and only if either of the two conditions holds

$$\begin{aligned} \Delta d_0 &< 0 \\ \Delta d_1 &> 0 \\ \Delta d_2 &< 0 \\ -2\Delta d_0 \Delta d_1 \Delta d_2 + 2(\Delta d_1)^3 - \Delta d_0(\Delta d_1)^2 - \Delta d_2(\Delta d_1)^2 + \Delta d_0(\Delta d_2)^2 + \Delta d_2(\Delta d_0)^2 &> 0 \end{aligned}$$

or

$$\begin{aligned} \Delta d_0 &> 0 \\ \Delta d_1 &< 0 \\ \Delta d_2 &> 0 \\ -2\Delta d_0 \Delta d_1 \Delta d_2 + 2(\Delta d_1)^3 - \Delta d_0(\Delta d_1)^2 - \Delta d_2(\Delta d_1)^2 + \Delta d_0(\Delta d_2)^2 + \Delta d_2(\Delta d_0)^2 &< 0 \end{aligned}$$

3 Games with n strategies

In the above section we assumed that each individual plays a pairwise game with its opponent. In addition, every individual can choose only between 2 strategies. In this section, we allow individuals to choose any number of strategies and thus generalise our analysis to n strategies. In this case, the pairwise interaction becomes an $n \times n$ game. We show that the previous results also hold for n strategies when the set dynamics are fast enough as, (i) the accumulated payoff for any strategy is an m-player game and (ii) the evolutionary dynamics of strategies can be captured by the replicator equation of the n-strategy m-player game.

3.1 Accumulated payoffs

Similar to the 2-strategy case, the breaking probability of a set depends exclusively on its strategy composition. Let us denote $k_{(\alpha_1, \alpha_2 \cdots, \alpha_n)}$ as the breaking probability of a set where α_s is the

number of strategy-s individuals in the focal set and $\alpha_s \geq 0$ and $\sum_{s=1}^{n} \alpha_s = m$ indicates that the set consists exactly of m individuals.

We start by randomly choosing one of the l sets, namely i. Then we define a sequence of sets T_i^t $(t \geq 0)$. Here the set T_i^t evolves into T_i^{t+1}. The type of the set T_i^t — i.e., $\psi(T_i^t)$ — is a Markov chain whose states are given by the possible set configurations. These set configurations can be denoted as the simplex

$$S_{n,m} = \{(\alpha_1, \alpha_2 \cdots, \alpha_n) | \alpha_s \geq 0 \text{ and } \sum_{s=1}^{n} \alpha_s = m\}, \quad [14]$$

where α_s is the number of strategy s individuals in the corresponding set. The transition matrix of this Markov chain is given by

$$Q = \frac{1}{l} V + \frac{l-1}{l} I, \quad [15]$$

where I is the identity matrix of size $|S_{n,m}|$. Here $|S_{n,m}|$ is the cardinal number of set $S_{n,m}$. V is the transition matrix conditioned on the fact that the set i is selected. By the updating rule of the sets, two subsequent sets T_i^t and T_i^{t+1} have at least $m-1$ individuals in common. Thus the transition is impossible between two states $(\alpha_1, \alpha_2 \cdots, \alpha_n)$ and $(\alpha'_1, \alpha'_2 \cdots, \alpha'_n)$, unless either of the following two cases holds.

- There exist two different strategies s_1 and s_2 such that $\alpha'_{s_2} = \alpha_{s_2} + 1$ and $\alpha'_{s_1} = \alpha_{s_1} - 1$; for all the other strategies s, $\alpha'_s = \alpha_s$.
- For all $1 \leq s \leq n$, $\alpha'_s = \alpha_s$.

In the first case, the selected set is broken; one individual playing strategy s_1 is expelled and one individual with strategy s_2 is incorporated to the set. In order to illustrate this case, we take the transition from $(\alpha_1, \alpha_2 \cdots, \alpha_n)$ to $(\alpha_1 + 1, \alpha_2 \cdots, \alpha_n - 1)$ as an example. First, a set consisting of α_s strategy s individuals is selected, and then breaks with probability $k_{(\alpha_1, \alpha_2 \cdots, \alpha_n)}$. Second, a strategy n individual is expelled (with probability α_n/m). Finally, a strategy 1 individual is incorporated (with probability x_A, i.e., the fraction of strategy 1 in the population). Thus the transition probability is $\alpha_n x_A k_{(\alpha_1, \alpha_2 \cdots, \alpha_n)}/m$. Similarly, the transition probability from state $(\alpha_1, \alpha_2 \cdots, \alpha_n)$

to $(\alpha_1', \alpha_2' \cdots, \alpha_n')$, where the two states fulfill the first constraint, is given by

$$\frac{\alpha_{s_1}}{m} x_{s_2} k_{(\alpha_1, \alpha_2 \cdots, \alpha_n)}. \quad [16]$$

The second case reveals that the two subsequent states are equivalent. Either the selected set is not broken or it is broken but the expelled individual and the new individual are the same in type. In this case, the transition probability can be obtained by the normalisation property of V — i.e., one minus the sum of all the other transition probabilities in Eq. (16).

When all the strategies coexist, i.e., $\Pi_{i=1}^n x_i \neq 0$, the transition matrix Q is aperiodic and irreducible, consequently the Markov chain presents a unique stationary distribution. By Eq. (15), the stationary distribution of Q is the same as that of V. This holds for any number of total links l. However, the size of the state space $|S_{n,m}|$ is $\binom{n+m-1}{m}$ (7). As a consequence, the number of states increases much more rapidly with the set size when there are more than two types of strategies in the population (Fig (2)). Given this, it becomes challenging to calculate the stationary distribution for multiple strategies. Still, as shown in (8), for general $n \times n$ games and the dynamical network $m = 2$, we have i) that the stationary distribution is a binomial distribution weighted by the duration time, ii) that the conditional transition matrix V satisfies the detailed balance condition. This binomial distribution arises from the network structure, which is a special case, $m = 2$, of our set structure. It turns out that these results can be generalised for $m \geq 2$.

- The stationary distribution of V, y, is a multinomial distribution weighted by the duration time, i.e.,

$$y_{(\alpha_1, \alpha_2, \cdots, \alpha_n)} = \frac{1}{\mathcal{N}} \frac{m!}{\alpha_1! \alpha_2! \cdots \alpha_n!} \frac{1}{k_{(\alpha_1, \alpha_2, \cdots, \alpha_n)}} \Pi_{i=1}^n x_i^{\alpha_i}, \quad (\alpha_1, \alpha_2, \cdots, \alpha_n) \in S_{n,m} \quad [17]$$

  where $\mathcal{N} = \sum_{(\alpha_1, \alpha_2, \cdots, \alpha_n) \in S_{n,m}} \frac{m!}{\alpha_1! \alpha_2! \cdots \alpha_n!} \frac{1}{k_{(\alpha_1, \alpha_2, \cdots, \alpha_n)}} \Pi_{i=1}^n x_i^{\alpha_i}$ is a normalisation factor.

- The Markov chain V fulfills the detailed balance condition, i.e.,

$$y_{(\alpha_1, \alpha_2, \cdots, \alpha_n)} V_{((\alpha_1, \alpha_2, \cdots, \alpha_n), (\alpha_1', \alpha_2', \cdots, \alpha_n'))} = y_{(\alpha_1', \alpha_2', \cdots, \alpha_n')} V_{((\alpha_1', \alpha_2', \cdots, \alpha_n'), (\alpha_1, \alpha_2, \cdots, \alpha_n))}. \quad [18]$$

We prove that the distribution Eq. (17) satisfies the detailed balance condition.

If the transition from state $(\alpha_1, \alpha_2, \cdots, \alpha_n)$ to state $(\alpha_1', \alpha_2' \cdots, \alpha_n')$ is impossible, then the reverse transition is also impossible. Thus, Eq. (18) holds. In the other cases, the transition is

possible. Therefore, the two states $(\alpha_1, \alpha_2, \cdots, \alpha_n)$ and $(\alpha'_1, \alpha'_2 \cdots, \alpha'_n)$ must satisfy one of the two constraints of the transition matrix.

If they fulfill the first constraint, i.e., there exist two different strategies s_1 and s_2 such that $\alpha'_{s_2} = \alpha_{s_2} + 1$ and $\alpha'_{s_1} = \alpha_{s_1} - 1$; for all the other strategies s, $\alpha'_s = \alpha_s$. By Eqs. (16) and (17) we have that

$$\begin{aligned} y_{(\alpha_1,\alpha_2,\cdots,\alpha_n)} V_{((\alpha_1,\alpha_2,\cdots,\alpha_n),(\alpha'_1,\alpha'_2,\cdots,\alpha'_n))} &= \tfrac{1}{\mathcal{N}} \tfrac{m!}{\alpha_1!\alpha_2!\cdots\alpha_n!} \tfrac{1}{k_{(\alpha_1,\alpha_2,\cdots,\alpha_n)}} \Pi_{i=1}^n x_i^{\alpha_i} \times \tfrac{\alpha_{s_1} x_{s_2} k_{(\alpha_1,\alpha_2,\cdots,\alpha_n)}}{m} \\ &= \tfrac{1}{\mathcal{N}} \tfrac{(m-1)!}{\alpha_1!\cdots(\alpha_{s_1}-1)!\cdots\alpha_{s_2}!\cdots\alpha_n!} \Pi_{i\notin\{s_1,s_2\}} x_i^{\alpha_i} \times x_{s_1}^{\alpha_{s_1}} x_{s_2}^{\alpha_{s_2}+1} \\ &= \tfrac{1}{\mathcal{N}} \tfrac{(m-1)!}{\alpha'_1!\cdots\alpha'_{s_1}!\cdots(\alpha'_{s_2}-1)!\cdots\alpha'_n!} \Pi_{i\notin\{s_1,s_2\}} x_i^{\alpha'_i} \times x_{s_1}^{\alpha'_{s_1}+1} x_{s_1}^{\alpha'_{s_2}} \\ &= \tfrac{1}{\mathcal{N}} \tfrac{m!}{\alpha'_1!\alpha'_2!\cdots\alpha'_n!} \tfrac{1}{k_{(\alpha'_1,\alpha'_2,\cdots,\alpha'_n)}} \Pi_{i=1}^n x_i^{\alpha'_i} \times \tfrac{\alpha'_{s_2} x_{s_1} k_{(\alpha'_1,\alpha'_2,\cdots,\alpha'_n)}}{m} \\ &= y_{(\alpha'_1,\alpha'_2,\cdots,\alpha'_n)} V_{((\alpha'_1,\alpha'_2,\cdots,\alpha'_n),(\alpha_1,\alpha_2,\cdots,\alpha_n))}. \end{aligned} \quad [19]$$

If they fulfill the second constraint, i.e., the two states are the same, then Eq. (18) holds naturally. Therefore the stationary distribution of Q is the multinomial distribution weighted by the duration time. Furthermore, Q fulfills the detailed balance condition.

When set dynamics are fast, the average payoff is determined by the stationary distribution of each set configuration. For any strategy $1 \le i \le n$, we have

$$\begin{aligned} f_i &= \frac{\text{Total payoff of strategy } i}{\text{Total number of strategy } i} \\ &= \frac{\sum_{\alpha\in S_{n,m}} (l y_\alpha) \left[\alpha_i \left(\sum_{j=1}^n a_{ij}(\alpha_j - \delta_{ij})\right)\right]}{N x_i}, \end{aligned} \quad (20)$$

where δ_{ij} is the Kronecker-delta and N is the population size.

Taking Eq. (17) into Eq. (20), we have f_i is given by

$$\frac{1}{\mathcal{N}} \frac{l}{N} \sum_{(\alpha_1,\alpha_2,\cdots,\alpha_n)\in S_{n,m}} \frac{m!}{\alpha_1!\alpha_2!\cdots\alpha_n!} \frac{1}{k_{(\alpha_1,\alpha_2,\cdots,\alpha_n)}} x_i^{\alpha_i - 1} \Pi_{k\neq i} x_k^{\alpha_k} \left[\alpha_i \left(\sum_{j=1}^n a_{ij}(\alpha_j - \delta_{ij})\right)\right]. \quad [21]$$

Considering that $\frac{m!}{\alpha_1!\cdots\alpha_i!\cdots\alpha_n!}\alpha_i = m\frac{(m-1)!}{\alpha_1!\cdots(\alpha_i-1)!\cdots\alpha_n!}$ yields that f_i is given by

$$\frac{1}{\mathcal{N}} \frac{lm}{N} \sum_{(\alpha_1,\alpha_2,\cdots,\alpha_n)\in S_{n,m}} \frac{(m-1)!}{\alpha_1!\cdots(\alpha_i-1)!\cdots\alpha_n!} x_i^{\alpha_i - 1} \Pi_{k\neq i} x_k^{\alpha_k} \frac{1}{k_{(\alpha_1,\alpha_2,\cdots,\alpha_n)}} \times \left(\sum_{j=1}^n a_{ij}(\alpha_j - \delta_{ij})\right) \quad [22]$$

Let $\tilde{\alpha}_k = \alpha_k - \delta_{ik}$, $(\tilde{\alpha}_1, \tilde{\alpha}_2, \cdots, \tilde{\alpha}_n)$ be the co-player configuration of a strategy i individual in a set $(\alpha_1, \alpha_2, \cdots, \alpha_n)$. Eq. (22) is given by

$$f_i = \frac{1}{\mathcal{N}} \frac{lm}{N} \sum_{(\tilde{\alpha}_1, \tilde{\alpha}_2, \cdots, \tilde{\alpha}_n) \in S_{n,m-1}} \underbrace{\frac{(m-1)!}{\tilde{\alpha}_1! \cdots \tilde{\alpha}_i! \cdots \tilde{\alpha}_n!} \Pi_{k=1}^{n} x_k^{\tilde{\alpha}_k}}_{\text{multinomial sampling}} \underbrace{\frac{1}{k_{(\tilde{\alpha}_1, \cdots \tilde{\alpha}_i + 1, \cdots, \tilde{\alpha}_n)}} \times \left(\sum_{j=1}^{n} a_{ij} \tilde{\alpha}_j \right)}_{\text{duration time} \times \text{collective payoff in the set}} \quad [23]$$

This accumulated payoff is formally equivalent to an n-strategy m-player game up to a rescaling factor $\frac{1}{\mathcal{N}} \frac{lm}{N}$. The first term is a multinomial distribution which indicates that $m-1$ co-players are sampled randomly as if in a well-mixed population. The second term shows that the payoff of strategy i of the multi-player game is the collective payoff of strategy i in a set times the average duration time of the corresponding set. This term is dependent on (i) the pairwise interaction between strategy i and (ii) the set duration time. This explicitly generates an n-strategy m-player game from a pairwise $n \times n$ game (a_{ij}).

3.2 Evolutionary dynamics of strategies

During the imitation process, the role model and the focal individual are both chosen randomly through the entire population. The evolution of strategies can also be approximated by the Langevin Equation. More precisely, in this case when the population is large enough, the demographic noise induced by the finite population size can be neglected (9). This results in the following replicator equation:

$$\dot{x}_i = x_i(f_i - \bar{f}), \quad [24]$$

where f_i is given by Eq. (23) and $\bar{f} = \sum_i^n x_i f_i$ is the average payoff. Consequently, the replicator equation is consistent with an n-strategy m-player game and can exhibit up to $(n-1)^{m-1}$ internal isolated equilibria (2).

Methods

Analytical calculation of the selection gradient.

The replicator equation associated to the Fermi process we apply in our simulation is given

by (10)

$$\dot{x}_A = x_A(1-x_A)\tanh\left[+\frac{\beta}{2}\left(f_A - f_B\right)\right], \qquad [25]$$

where $f_A = \frac{lm}{NN}\tilde{f}_A$ and $f_B = \frac{lm}{NN}\tilde{f}_B$. The rescaled selection gradient $\dot{x}_A/(x_A(1-x_A))$ is thus given by the hyperbolic tangent of the payoff difference, scaled by the intensity of selection,

$$\tanh\left[+\frac{\beta}{2}\left(f_A - f_B\right)\right]. \qquad [26]$$

Numerical simulation of the selection gradient.

Each data point is the average of 100 independent realisations. Every realisation takes 10^7 generation. For the first 10^4 generations of each realisation, only set dynamics occur. After that, with a probability of $w = 10^{-3}$ two individuals are chosen randomly from the entire population. The first individual is the "focal" one which is the one that may imitate the strategy of the second one based on the Fermi rule. We keep track of the transition without implementing them to infer the strength and direction of selection. We denote s_A and s_B as the number of times that an individual playing strategy A and B switches to the other strategy. $\frac{s_B - s_A}{Q}$ is our estimator of the selection gradient $\dot{x}_A$, where Q is the number of strategy updating events in this realisation.

References

[1] Blume, A. Communication, risk, and efficiency in games. *Games and Economic Behavior* **22**, 171–202 (1998).

[2] Gokhale, C. S. and Traulsen, A. Evolutionary games in the multiverse. *Proceedings of the National Academy of Sciences USA* **107**, 5500–5504 (2010).

[3] Traulsen, A., Pacheco, J. M., and Imhof, L. A. Stochasticity and evolutionary stability. *Physical Review E* **74**, 021905 (2006).

[4] Wu, B., Altrock, P. M., Wang, L., and Traulsen, A. Universality of weak selection. *Physical Review E* **82**, 046106 (2010).

[5] Farouki, R. T. The Bernstein polynomial basis: A centennial retrospective. *Computer Aided Geometric Design* **29**, 379–419 (2012).

[6] Peña, J., Lehmann, L., and Nöldeke, G. Gains from switching and evolutionary stability in multi-player matrix games. *Journal of Theoretical Biology* **346**, 23–33 (2014).

[7] Reingold, E. M., Nievergelt, J., and Deo, N. *Combinatorial algorithms: theory and practice*. Englewood Cliffs, N.J. : Prentice-Hall, cop., (1977).

[8] Wu, B., Zhou, D., and Wang, L. Evolutionary dynamics on stochastic evolving networks for multiple-strategy games. *Physical Review E* **84**(046111) (2011).

[9] Traulsen, A., Claussen, J. C., and Hauert, C. Coevolutionary dynamics: From finite to infinite populations. *Physical Review Letters* **95**, 238701 (2005).

[10] Traulsen, A., Nowak, M. A., and Pacheco, J. M. Stochastic dynamics of invasion and fixation. *Physical Review E* **74**, 011909 (2006).

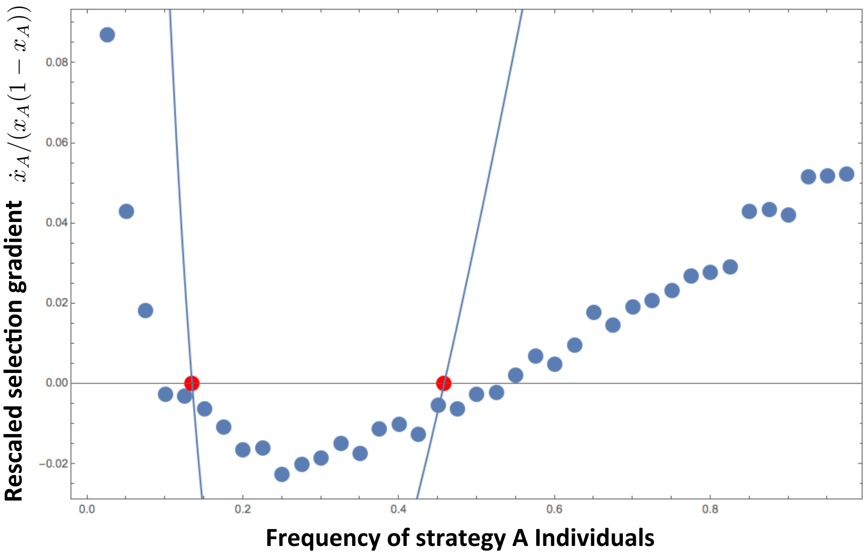

Figure 1: The rescaled selection gradient $\dot{x}_A/(x_A(1-x_A))$ captures the strength and direction of selection. A simulation based on the Fermi update rule (dots, see Methods) shows that the selection gradient has two roots. The analytical approximation (Eq.(10)) agrees with the equilibria of the selection gradient obtained by simulation $x_A^* \approx 0.13$ and 0.46 (red dots). The absolute value of the selection gradient, however, is systematically overestimated by the analytical approximation. This is due to the convexity of the hyperbolic tangent. Let us assume that $Q(f_A^*, f_B^*)$ is the probability that a strategy A individual has the payoff f_A^* and a strategy B individual has the payoff f_B^*. Then, the selection gradient based on simulation is an estimator of $\sum_{f_A^*, f_B^*} \tanh(\frac{\beta}{2}(f_A^* - f_B^*))Q(f_A^*, f_B^*)$. Since $\tanh(x)$ is convex for $x > 0$, thus the analytical approximation $\tanh(\frac{\beta}{2}(f_A - f_B)) = \tanh(\sum_{f_1^*, f_2^*} \frac{\beta}{2}(f_1^* - f_2^*)Q(f_1^*, f_2^*))$ is greater than the estimator of the simulation $\sum_{f_A^*, f_B^*} \tanh(\frac{\beta}{2}(f_A^* - f_B^*))Q(f_A^*, f_B^*)$. By similar arguments, we obtain that the analytical approximation underestimates the simulation result for negative selection gradient. Each blue dot in the plot is the average of 100 independent realisations. Every realisation takes 10^7 generation. For the first 10^4 generations of each realisation, only set dynamics occur. After that, with a probability of $w = 10^{-3}$ two individuals are chosen randomly from the entire population. We keep track of the transition without implementing them. We denote y and z as the number of times that an individual playing strategy A and B changes its strategy. $\frac{z-y}{Q}$ is the estimator of the selection gradient $\dot{x_A}$, where Q is the number of strategy updating events in this realisation. (Parameters: Stag-Hunt game with $a_{AA} = 2$, $a_{AB} = 1$, $a_{BA} = 1.5$ and $a_{BB} = 7$. Population size, $N = 500$, number of sets, $l = 1000$, probability of a strategy update, $w = 10^{-3}$. Selection intensity, $\beta = 0.1$. The breaking probabilities are $k_i = (1 + 10i)^{-1}$, where i is the number of strategy A individuals in the set.)

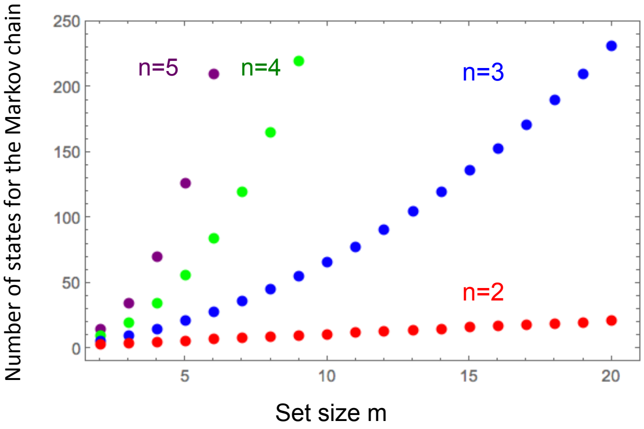

Figure 2: The size of the state space of the Markov chain of the set dynamics as a function of the set size m. For a two-strategy game, there are $m+1$ set configurations. For a three-strategy game, there are $\binom{m+2}{2} = \frac{(m+2)(m+1)}{2}$ set configurations. In general, the number of the states increases rapidly with the size of the set, if the strategy number increases.
